# Development of machine learning models for patients in the high intrahepatic cholangiocarcinoma incidence age group

**DOI:** 10.1186/s12877-024-05154-3

**Published:** 2024-06-25

**Authors:** Jie Shen, Dashuai Yang, Yu Zhou, Junpeng Pei, Zhongkai Wu, Xin Wang, Kailiang Zhao, Youming Ding

**Affiliations:** 1 Dept of hepatobiliary surgery, Renmin Hospital of Wuhan University, Wuhan, Hubei 430060 China; 2 Dept of hepatobiliary surgery, 521 Hospital of Norinco Group, Xi’an, Shaanxi 710061 China

**Keywords:** Intrahepatic cholangiocarcinoma, High-incidence age, Machine learning, Random survival forest, Prognostic system

## Abstract

**Background:**

Intrahepatic cholangiocarcinoma (ICC) has a poor prognosis and is understudied. Based on the clinical features of patients with ICC, we constructed machine learning models to understand their importance on survival and to accurately determine patient prognosis, aiming to develop reference values to guide physicians in developing more effective treatment plans.

**Methods:**

This study used machine learning (ML) algorithms to build prediction models using ICC data on 1,751 patients from the SEER (Surveillance, Epidemiology, and End Results) database and 58 hospital cases. The models’ performances were compared using receiver operating characteristic curve analysis, C-index, and Brier scores.

**Results:**

A total of eight variables were used to construct the ML models. Our analysis identified the random survival forest model as the best for prognostic prediction. In the training cohort, its C-index, Brier score, and Area Under the Curve values were 0.76, 0.124, and 0.882, respectively, and it also performed well in the test cohort. Kaplan–Meier survival analysis revealed that the model could effectively determine patient prognosis.

**Conclusions:**

To our knowledge, this is the first study to develop ML prognostic models for ICC in the high-incidence age group. Of the ML models, the random survival forest model was best at prognosis prediction.

**Supplementary Information:**

The online version contains supplementary material available at 10.1186/s12877-024-05154-3.

## Introduction

The incidence and mortality rates of intrahepatic cholangiocarcinoma (ICC), which is the second most common primary liver cancer, accounting for 10% of all primary liver cancers, are increasing [1]. When compared with hepatocellular carcinoma (HCC), ICC is less well understood and also has a worse prognosis. Although radical surgery is a curative treatment for patients with early-stage ICC, many patients are diagnosed at an advanced stage. Moreover, a large-capacity center study found that after hepatectomy, patients with ICC have a five-year survival rate of 25–35%, which was mainly attributed to a high recurrence rate [2].

Although the American Joint Committee on Cancer (AJCC) staging is the most widely used system of evaluating the prognosis of patients with ICC, it is less accurate because it does not account for the effects of treatment, age, and other important factors [3]. Although nomograms have been increasingly researched in recent years [4, 5], they are based on multivariate Cox regression analysis with fixed assigned weights, which are outdated and rigid tools [6]. Through machine learning (ML) algorithms, computers can learn from large-scale, disparate healthcare data and then make decisions or predictions without being explicitly programmed. In many tasks, such as diagnosis, classification, and survival prediction, ML models have key advantages over traditional statistical models [7].

According to the 2020 Global Cancer Observatory (Cancer Today [iarc.fr]), the number of liver cancer cases rose sharply after the age of 50 years, while its incidence fell after the age of 74 years. Therefore, for ICC, patients aged between 50 and 74 years are the most frequent and representative. Focusing on this group, we investigated the impact of the clinical features of patients with ICC on survival and accurate prognosis based on ML algorithms, aiming to provide reference values for guiding clinicians in making treatment plans.

## Methods

### Patient selection and study variables

**Fig. 1:**
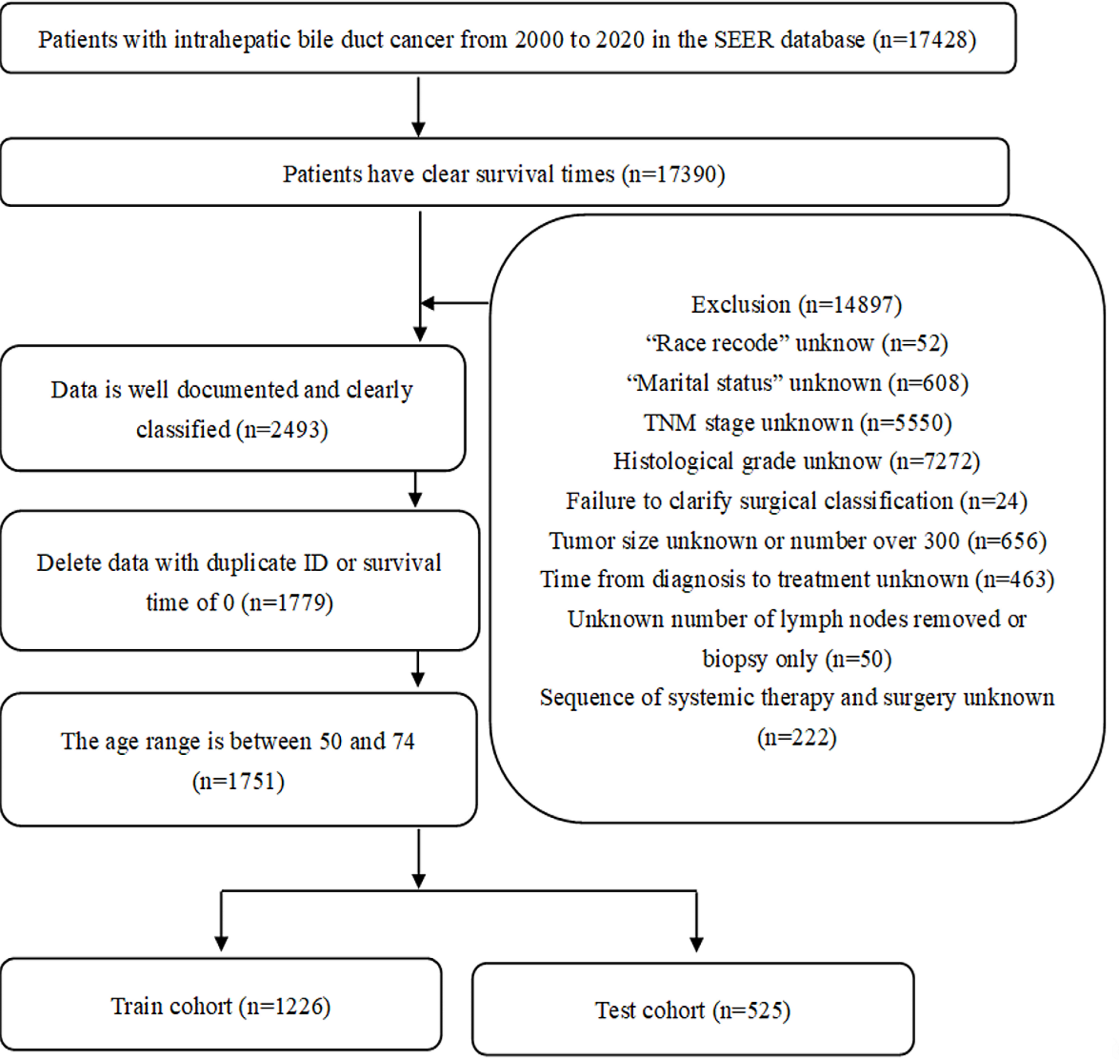
Flow chart of patients’ selection in the training and test cohorts from the SEER database

Data on patients with ICC, who were diagnosed with primary intrahepatic bile duct cancer between 2000 and 2020, were obtained using SEER*Stat software (version 8.4.2). External validation data, with diagnosis supported by clear pathology results, were obtained from Renmin Hospital of Wuhan University. The study’s ethical approval was granted by the Clinical Research Ethics Committee, Renmin Hospital of Wuhan University. Study variables included age, sex, race, marital status, time between diagnosis and treatment, histological grade, AJCC-TNM stage, tumor site surgery information, regional lymph node removal information, tumor size, sequence number, number of tumors (number of malignant tumors in lifetime), sequence of surgery and systemic therapy, chemotherapy, radiotherapy. The data of patients with the primary site code, C22.1, an age of 50–74 years, and complete follow-up information were included. Those with missing or unclear data records, controversial grouping data, and a survival of less than a month, were excluded. In case two or more medical records were available, the most recent one prevailed. The patient screening process is outlined in Fig. 1.

### Variable selection and machine learning model construction

Data from 1,751 patients with ICC were randomly divided at a 7:3 ratio into training and internal test cohorts. Univariate and multivariate Cox analyses were then used to identify variables with prognostic value (statistically significant variables with hazard ratios [HR] of > 1 or < 1). The prediction models were constructed using the open-source package, Python library scikit-survival, version 0.21.0 (Python version 3.11.4) [8].

### Evaluation of model prediction accuracy and superiority

C-index, time-dependent AUC, and Brier score analyses were used to assess model prediction accuracy [9, 10]. The Brier score measures the difference between the predicted probability and the true outcome, with higher scores indicating poorer prediction accuracy and calibration [11]. The ML models were compared and analyzed using decision curve analysis (DCA). The best cutoff value for risk grouping was determined using the X-tile software [12]. The patients were then classified into the high-, medium-, or low-risk groups. The differences in the groups’ overall survival (OS) rates and actual patient survival probabilities were determined using Kaplan–Meier (KM) analysis.

### Interpretation of the random survival forest (RSF) model

The model’s interpretation was divided into the SHapley Additive exPlanations (SHAP) plot and the JAVA-based prediction website. SHAP, a model interpretation package developed in Python, is used for ML model interpretation. For each prediction sample, a SHAP value is assigned to each feature, and the larger the absolute SHAP value, the greater the feature’s influence. The value’s sign indicates if the feature affects the result positively or negatively [13, 14]. To improve this study’s practical value, an interactive website was developed, on which one-, three-, and five-year OS can be calculated automatically by entering the required clinical information.

### Statistical analyses

Statistical analyses were performed on R version 4.2.1 and Python version 3.11.4. The “survival”, “survminer”, and timeROC” packages were used for univariate and multivariate Cox regression, forest mapping, and receiver operating characteristic (ROC) analyses, respectively. HR > 1 and < 1 indicate risk and protective factors, respectively. A Chi-square test was used to assess distribution differences of the variables in the two cohorts. Survival rates were compared using a log-rank test. All statistical tests were two-sided, with *P* < 0.05 indicating statistically significant differences.

## Results

### Baseline characteristics of the training and test cohorts

The study involved 1,751 patients (women: 830) with ICC from the SEER database, who were divided into the training (*N* = 1226, 70%) and test (*N* = 525, 30%) cohorts. More than half of the patients started treatment within a month of diagnosis, and almost all were treated within three months. Although the number of patients with various TNM stages was about the same, the histological grade of the tumors was mainly moderately or poorly differentiated, and highly differentiated or undifferentiated tumors were less common. Many studies indicate that surgery is the main treatment strategy for ICC [15, 16]. In the training cohort, most patients underwent hepatectomy, including wedge or segmental resection, lobectomy, extended lobectomy, hepatectomy, and bile duct excision. Very few patients received liver transplantation or local tumor destruction, such as cryotherapy, photodynamic therapy, and radiofrequency ablation. However, 33% of the patients did not undergo surgery. Notably, most patients also received chemotherapy. The training and test cohorts’ baseline data are shown in Table 1.

**Table 1:** Demographic and clinical characteristics of patients with intrahepatic cholangiocarcinoma

Variable	Overall		Train cohort		Test cohort		*P* Value
	Sample size	Percentage	Sample size	Percentage	Sample size	Percentage	
Total	1751		1226		525		
**Sex**							
female	830	47.40%	583	47.55%	247	47.05%	0.89
male	921	52.60%	643	52.45%	278	52.95%	
**Grade** ^a^							
I	187	10.68%	136	11.09%	51	9.72%	0.39
II	869	49.63%	594	48.45%	275	52.38%	
III	682	38.95%	488	39.80%	194	36.95%	
IV	13	0.74%	8	0.65%	5	0.95%	
**AJCC Stage**							
I	527	30.10%	364	29.69%	163	31.05%	0.29
II	370	21.13%	264	21.53%	106	20.19%	
III	257	14.68%	169	13.79%	88	16.76%	
IV	597	34.09%	429	34.99%	168	32%	
**Diagnosis to treat**							
0 ∼ 1	1024	58.48%	715	58.32%	309	58.86%	0.92
2 ∼ 3	590	33.70%	413	33.69%	177	33.71%	
> 3	137	7.82%	98	7.99%	39	7.43%	
**Surgery**							
no surgery	585	33.41%	410	33.44%	175	33.33%	0.66
local treatment	33	1.88%	25	2.04%	8	1.52%	
hepatectomy	1090	62.25%	764	62.32%	326	62.10%	
transplantation	43	2.46%	27	2.20%	16	3.05%	
**Tumor size**							
< 5 cm	715	40.83%	509	41.52%	206	39.24%	0.43
5–10 cm	773	44.15%	541	44.13%	232	44.19%	
> 10 cm	263	15.02%	176	14.36%	87	16.57%	
**Chemotherapy**							
yes	1129	64.48%	789	64.36%	340	64.76%	0.91
no/unknown	622	35.52%	437	35.64%	185	35.24%	
**Sequence number** ^**b**^							
One primary only	1270	72.53%	890	72.59%	380	72.38%	0.94
1st of 2 or more	108	6.17%	74	6.04%	34	6.48%	
not 1st primary	373	21.30%	262	21.37%	111	21.14%	

^a^ “Grade” refers to histological grade. In pathological reports, highly differentiated ICC corresponds to “Grade I”, moderately differentiated ICC corresponds to “Grade II”, poorly differentiated ICC corresponds to “Grade III”, and undifferentiated ICC corresponds to “Grade IV”

^b^ “Sequence number” refers to the order in which the cancers in a patient’s lifetime compared to ICC. “One primary only” means that the patient has only ICC in his or her lifetime. “1st of 2 or more” means that the ICC is the patient’s first malignant tumor, but later developed other tumors. “not 1st primary” means that the patient had other tumors prior to ICC

### Variable selection

**Fig. 2:**
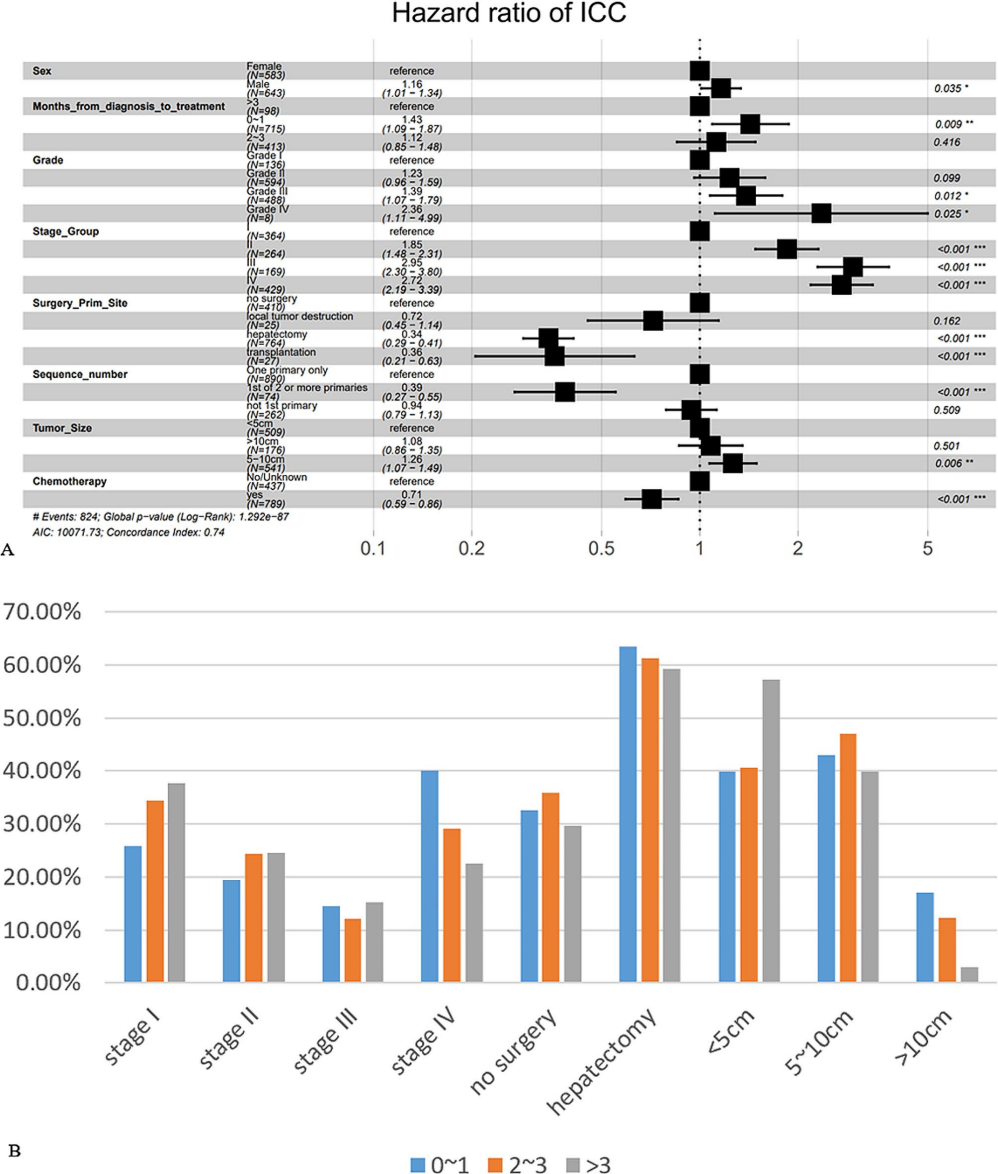
Demonstration of multivariate Cox analysis and analysis of different months from diagnosis to treatment. **A** Forest plot based on multivariate Cox regression analysis. **B** Bar plot of important features of ICC patients in different months from diagnosis to treatment. The vertical coordinate is the percentage of the feature subgroup in the group

Cox regression analyses were used to identify prognostic variables in the training cohort. The study included 15 variables and after univariate Cox analysis, the variables, age, race, marital status, and radiotherapy were excluded (Table S1). This analysis was followed immediately by a multivariate Cox analysis (Table S2). analysis revealed that the number of malignant tumors and sequence numbers had a high degree of collinearity problem. Therefore, only the malignant tumors’ sequence number was retained. Finally, eight statistically significant prognostic factors were selected (Fig. 2A).

As shown in the figure, when compared with women, OS was slightly worse in men, which is consistent with previous reports on HCC and most other cancers [17, 18]. Surprisingly, in the group in which the time between diagnosis and treatment was less than one month, the HR was higher than in the group in which the time was over three months, which is counterintuitive and contrary to several reports [19]. We therefore hypothesized that this feature was overshadowed by other important features because of a small sample size and conducted a correlation analysis. In group “0–1 month” (Fig. 2B), the proportion of TNM stage I was significantly lower than stage IV, whereas the opposite trend was observed in patients in the group, “>3 months”, with the “>3 months” group having a significantly higher percentage of stage I patients when compared with the other two groups. This analysis also revealed that most tumors in the “>3 months” group were < 5 cm in size, whereas those in the “0–1 month” group had a significant number of tumors that were > 10 cm in size.

Histological grade, AJCC-TNM stage, and tumor size correlated negatively with OS and unsurprisingly, surgery and chemotherapy were more beneficial to prolong OS, with hepatectomy and liver transplantation (LT) being significantly better than local tumor destruction. However, the analysis did not reveal the advantages of LT over hepatectomy, probably because LT data were available for only about 2% of the cases, large individual differences may affect outcomes. Interestingly, patients in “1st of 2 or more” had relatively more optimistic prognoses than patients with ICC only. However, there were no significant differences when compared with “not 1st primary”.

### ML model construction and comparison

**Fig. 3:**
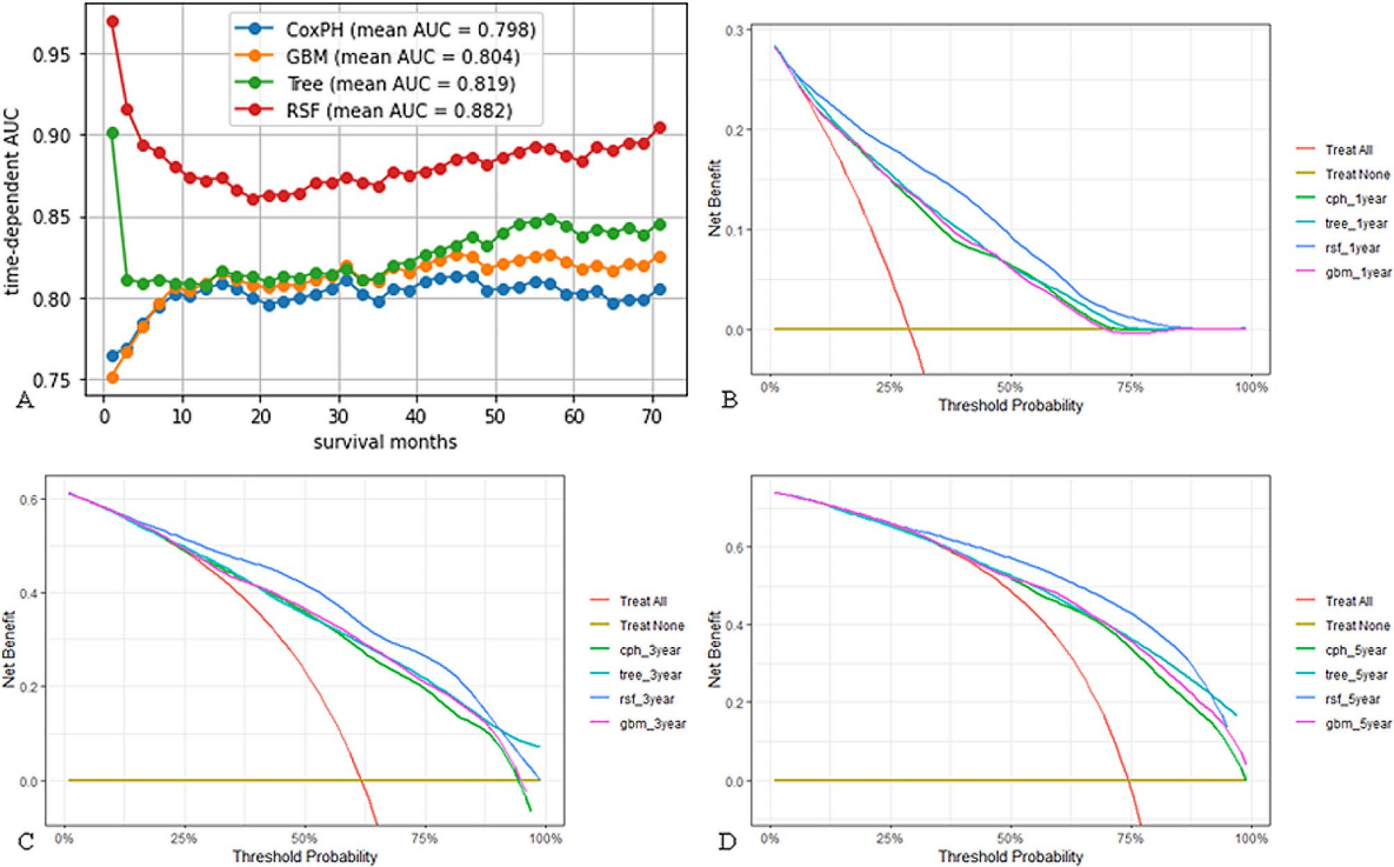
Evaluation of the performance of four ML models. **A** Time-dependent AUC for the four models. **B**-**D** DCA of ML models for one-year, three-year, and five-year OS prediction in the training cohort

Cox proportional hazards (CPH), survival tree (Tree), gradient boosted machine (GBM), and RSF models were developed based on the training cohort and their parameters were optimized using a five-fold crossover (Table S3 and Fig. S1). To evaluate the models’ performances, their C-indexes and Brier scores were first calculated (Table 2). These analyses revealed that the RSF model performed best, with a high C-index (0.76) and a low Brier score (0.124). Next, we calculated the four models’ Area Under the Curve (AUC) values over time (Fig. 3A). This analysis revealed that for the RSF model, the average AUC value was 0.882, which was markedly higher than the AUCs of other models. Importantly, the RSF model’s AUC value in the first year was higher than in the other periods, indicating that it could predict short-term prognosis more accurately. DCA revealed that the use of our models, especially the RSF model, to guide treatment can benefit patients (Fig. 3B–D). Because the RSF model performed much better than the other three models, it was used for follow-up analyses.

**Table 2:** C-index and Brier score of machine learning models

	C-index	Brier Score
RSF	0.76	0.124
GBM	0.745	0.150
CPH	0.739	0.154
Tree	0.735	0.145
Random	0.500	0.251

### Validation of the RSF model’s performance

**Fig. 4:**
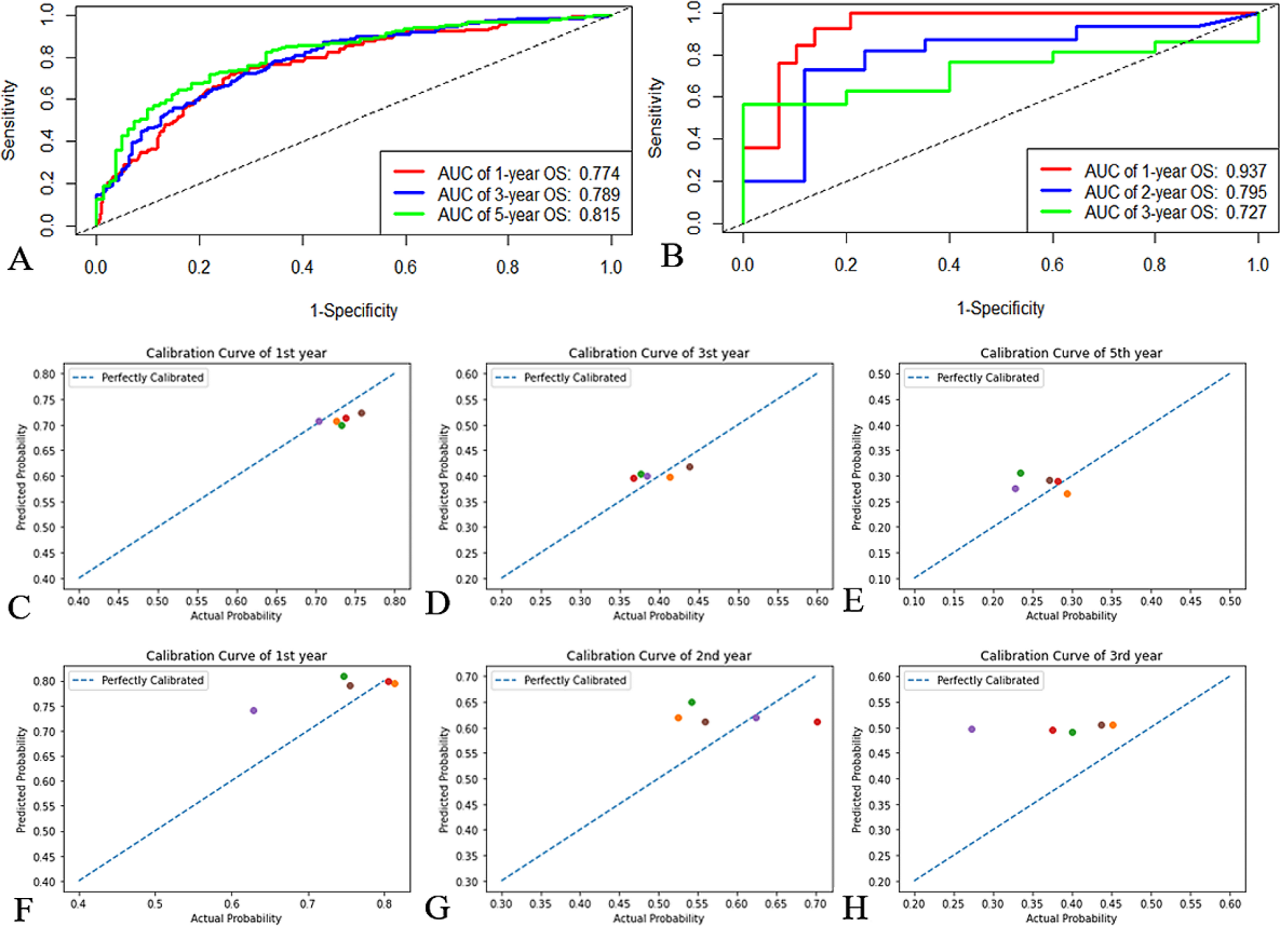
ROC curves and calibration curves of the RSF model in test cohorts. **A** ROC curves for RSF model predicting 1-, 3-, and 5-year OS in the internal test cohort. **B** ROC curves for RSF model predicting 1-, 2-, and 3-year OS in the external test cohort. **C**, *D*, *E* Calibration curves of first **C**, third **D** and fifth **E** year in the internal test cohort. **F**, *G*, *H* Calibration curves of first **F**, second **G** and third **H** year in the external test cohort

The RSF model’s performance was validated in internal and external test cohorts. The external test cohort had 58 patients (S2). The RSF model performed well in both cohorts (both C-indexes: >0.72, both Brier scores: <0.18, Table S4). In the internal test cohort, ROC curve analysis revealed that the model’s AUC values for one-, three-, and five-year OS were 0.774, 0.789, and 0.815, respectively. However, because of an insufficient sample size, fifth-year ROC curve analysis could not be conducted on the external test cohort. The analysis was therefore done for the second year. Surprisingly, in the external test cohort, the model’s predictive accuracy was high in the first year (AUC: 0.937), and it also performed well in the second (AUC: 0.795) and third years (AUC: 0.727). The model was further evaluated by comparing the consistency between actual survival probabilities and the predicted probabilities (Fig. 4C–F). This analysis revealed that the model’s predictions were highly consistent with the actual situation.

### Risk stratification based on the RSF model

**Fig. 5:**
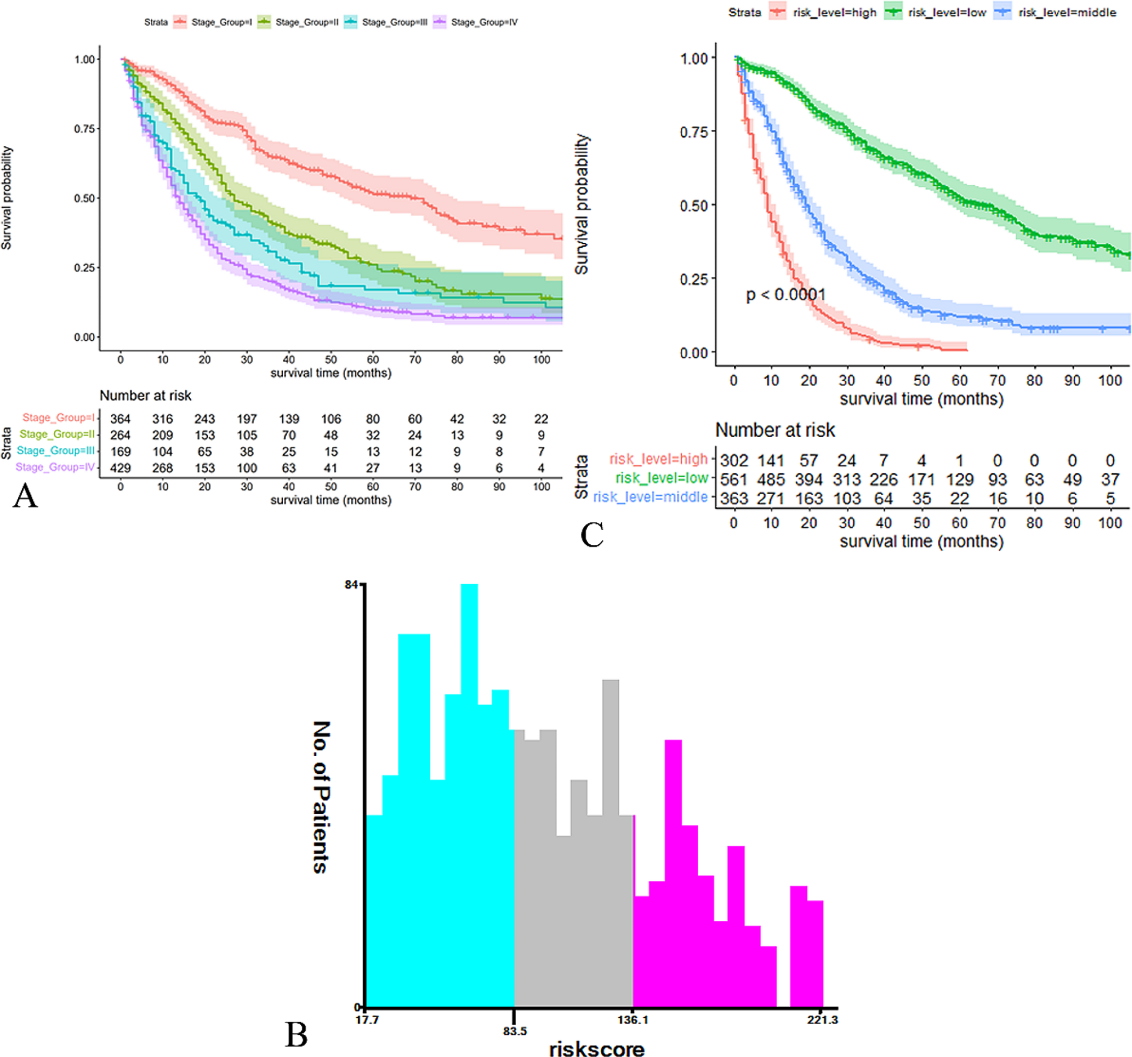
Risk stratification system based on RSF model. **A** Survival curves based on TNM stage. **B** Cut off values for optimal grouping determined using X-tile. **C** KM survival curves based on RSF model

The ability of TNM staging to predict patient prognosis was poor (Fig. 5A). We therefore developed a risk stratification system based on the training cohort’s patient risk scores (Fig. 5B). Patient risk scores were determined from the RSF model’s predictions and they ranged from 17.7 to 221.3, with scores of < 83.5 indicating low risk, scores of > 136.1 indicating high risk, and scores that fall between these values indicating intermediate risk. KM analysis revealed that patients in various subgroups had significantly different OS rates (Fig. 5C), with the high-risk group having the worst prognosis and the low-risk group having the best prognosis.

### The RSF model’s feature importance and interpretation

**Fig. 6:**
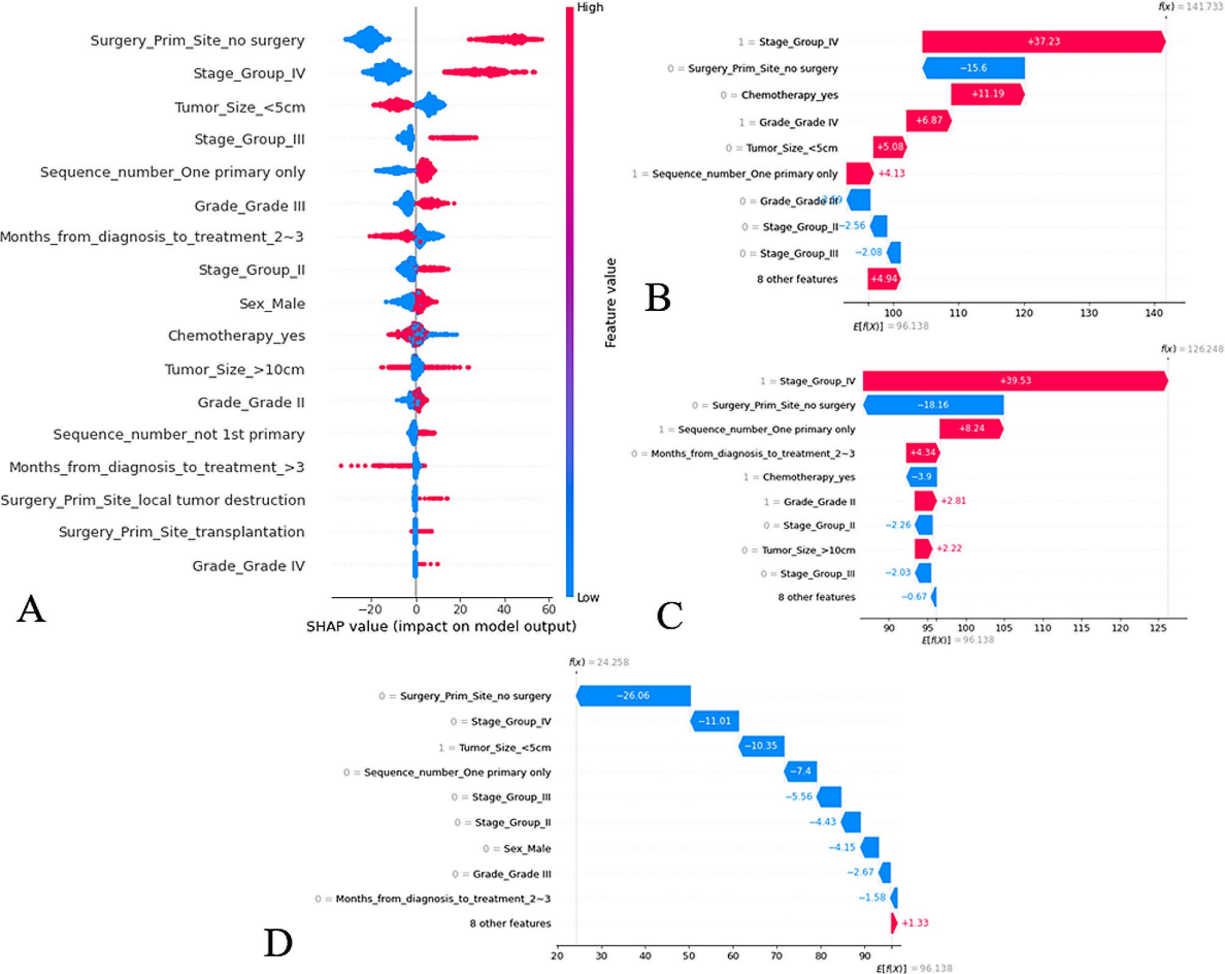
The SHAP plot of the RSF model. **A** SHAP beeswarm summary plot on the impact of input variables on the RSF model’s prediction. **B** The local SHAP plot of the patient #1. **Patient #1**: 50-year-old male, survival time was 1 month, died. AJCC TNM stage was IV, Histological grade was IV, tumor size = 12.5 cm. He was treated immediately after diagnosis, underwent hepatectomy and chemotherapy, only had intrahepatic cholangiocarcinoma in his life. **C** The local SHAP plot of the patient #2. **Patient #2**: 66-year-old female, survival time was 45 months, died. AJCC TNM stage was IV, Histological grade was II, tumor size = 2.0 cm. She was treated 1 month after diagnosis, underwent hepatectomy and chemotherapy, only had ICC in his life. **D** The local SHAP plot of the patient #3. **Patient #3**: 66-year-old female, survival time was 18 months, alive. AJCC TNM stage was I, Histological grade was II, tumor size = 3.2 cm. She was treated 1 month after diagnosis, underwent hepatectomy and chemotherapy. Prior to being diagnosed with ICC, she had multiple malignant tumors. The red ribbons in the local SHAP plot represent risk factors that lead to a poor prognosis, whereas the blue ribbons are the relatively protective factors

The SHAP technique calculates each feature’s contribution to the model’s final prediction decision for any instance, x_i_. In the SHAP figure (Fig. 6), the model’s variables are listed in descending order based on importance, with the variable, ‘whether the tumor primary site underwent surgery’, being the most important. Positive SHAP values indicated an increased probability of “death”, with higher values indicating higher risk and vice versa. The results indicate that ‘no surgery at the tumor primary site’ and TNM stage IV increased the probability of “death”, whereas a tumor size of < 5 cm increased the probability of “survival”. To demonstrate prognosis prediction, three patients were randomly selected from the training cohort (Fig. 6B–D). To present our model more intuitively and facilitate its use by clinicians, we developed a website (http://39.101.130.191:8888/icca) where users can predict OS by entering their data and then clicking “determine” to get the predicted results. The model can also be used to assess if a treatment is beneficial. By controlling for the same ‘other variables’ and then inputting a different treatment, one can assess if the prediction improves or decreases, thereby determining if an intervention is beneficial.

## Discussion

Despite liver cancer incidence and mortality increasing annually, accurate prognostic models for guiding clinical decisions are lacking since most current models are Cox regression-based nomograms. Here, we found that ICC incidence is highest in the 50–74 years age group and sought to develop CPH, Tree, GBM, and RSF models for predicting ICC prognosis in this age group [20]. Our findings indicate that ML models exhibit good predictive performance, with the RSF model exhibiting the highest prognosis prediction accuracy. In the training cohort, the RSF model had a C-index of 0.76, a Bries score of 0.124, and an average AUC value of 0.882. Further validation analysis of the model’s utility and accuracy using internal and external tests revealed C-indexes of 0.72 (internal) and 0.80 (external).

Based on Cox regression analyses, our model incorporated eight variables. In most cancers, women have better OS than men [17, 18]. Cong et al. suggested that this may be because women have a better liver foundation since only about 49% of women with liver cancer have cirrhosis when compared to 68% of men [21]. Other studies indicate that the longer OS may be because of earlier liver cancer detection since more women undergo regular ultrasound and α-fetoprotein (AFP) tests, and therefore have better treatment results [22]. However, it is also reported that molecular factors may account for gender differences in OS, such as differential CXCL14, ATF5HAMP, and GPR37 expression, and different levels of Notch and PI3K/AKT signaling [23]. Regarding how the time between diagnosis and treatment affects prognosis, there are discrepancies in reported studies. One study reported that delay in the time from diagnosis to treatment did not significantly affect the OS of patients with liver cancer [24]. However, Tsai et al. reported that in early liver cancer, the longer the time between diagnosis and treatment, the lower the survival rate [25]. Interestingly, some studies indicate that the time interval between diagnosis and treatment may not correlate significantly with prognosis and that it may correlate with prognosis positively or negatively [26]. Therefore, the effect of this factor on ICC prognosis remains controversial.

Histologically, ICC is a highly-to-moderately differentiated adenocarcinoma that in the early stages, often invades the portal vein, lymphatic vessels, and intrahepatic nerves [27]. Moreover, the larger the tumor, the higher the vascular invasion incidence, and in many cancers, tumor size is a prognostic factor [28, 29]. Tumors with a size of ≤ 2 cm have been associated with a good five-year survival rate (63.4%), while patients with tumors that are ≤ 2 cm and no lymph node metastasis, portal vein invasion, or biliary ductal invasion, have a 100% five-year survival rate. However, tumors with a size of > 2 cm are associated with a decline in the five-year survival rate [30]. Although tumor size affects prognosis, resection indications are not limited to tumor size.

Our results indicate that the AJCC-TNM stage and surgery at the tumor site are the most important factors affecting OS. It is reported that at the time of diagnosis, only 20–30% of patients are eligible for resection, mainly because of multifocal tumors and metastases [31]. The main surgical intervention for ICC is hepatectomy, which offers patients about three years of disease-free survival. For advanced, localized, or unresectable ICC, local treatments like transcatheter arterial chemoembolization and thermal ablation are widely used, which, as palliative care, can significantly prolong OS [32]. Importantly, for tumor sizes of < 3 cm, thermal ablation is reported to have a similar impact on survival as hepatectomy and a lower complication rate while being less expensive [33]. LT efficacy in ICC is reported to be significantly worse than that of HCC [34, 35]. However, although LT is often a treatment option for unresectable malignant liver and bile duct tumors, our findings do not show LT’s superiority over hepatectomy because of an insufficient amount of data. However, a recent study reported satisfactory LT results showing that in carefully selected patients with ICC, when combined with neoadjuvant chemotherapy, LT resulted in a five-year OS rate of 83.3% and a five-year disease-free survival of 50% [36]. Therefore, for ICC, it is important to identify ideal LT candidates, and further research is needed.

Over the past decade, gemcitabine and cisplatin have become the standard postoperative adjuvant ICC therapy. It is also reported that neoadjuvant therapy can benefit the survival of patients with ICC [37, 38]. Other studies have shown that surprisingly, combining trans-arterial drug-eluting bead therapy with chemotherapy was efficacious [31, 39]. It is interesting to note the effect of the variable, ‘sequence number’, on ICC prognosis in this study. Although we did not identify relevant ICC studies, a study by Heo et al. found that in HCC, patients with cancer and longer survival had a higher risk of developing a second primary tumor, indicating that patients who developed a second primary tumor survived relatively longer [40]. Similar results were reported by Wang et al. for small cell lung cancer [41], who showed that patients with lung cancer (LC) may die prematurely because of poorer health or higher tumor malignancy, without suffering from other tumors. Moreover, patients with LC, who develop additional tumors, inevitably receive additional antitumor therapy, which may also act as anti-LC therapy. Finally, patients with simple LC may have defective immune surveillance, which may lead to “immune escape”, whereas secondary tumors may activate cancer-related immune mechanisms. These factors may also apply to ICC.

Our research is progressive. Using the latest SEER data, we first used ML algorithms to construct prognostic models for the high ICC incidence age group. We have overcome the visualization and application challenges of ML models using the SHAP technique and by developing a prediction website. However, this study has limitations. First, because it is retrospective, it may have selection bias. Therefore, prospective studies are needed to validate our findings. Second, the SEER database only covers the U.S., and the external test cohort used in this study had 58 patients only. This study would have been more robust if it involved larger datasets. Because of SEER database limitations, some potentially important variables, such as targeted therapy, immunotherapy, and genetic factors, were not available, and including them may improve the performance of ML models.

## Conclusion

This study used eight variables to construct ML models for predicting the prognosis of patients in the high ICC incidence age group. Our analyses indicate that the RSF model could predict ICC OS most accurately.

### Electronic supplementary material

Below is the link to the electronic supplementary material.

Supplementary Material 1

Supplementary Material 2

Supplementary Material 3

## Data Availability

The dataset used in this study can be requested from the SEER source website at https://seerdataaccess.cancer.gov/seer-data-access.
