# Supplementary material for: Development of machine learning models for patients in the high intrahepatic cholangiocarcinoma incidence age group
Source: BMC Geriatr. 2024 Jun 25;24:553. doi: 10.1186/s12877-024-05154-3 (PMC11197277; doi:10.1186/s12877-024-05154-3)
Supplement: Supplementary file 1 — Supplementary Material 1 [file 12877_2024_5154_MOESM1_ESM.docx]

**Appendix S1**

**Table S1:**

**Univariate cox regression analysis of the training cohort.**

| **Characteristics** | **HR** | **95%CI** | **P Value** | |
| --- | --- | --- | --- | --- |
| Sex |  |  |  | |
| female | reference |  | |  |
| male | 1.16 | 1.01-1.33 | | 0.04 |
| TNM Stage |  |  |  |  |
| I | reference |  | |  |
| II | 2 | 1.61-2.49 | | <0.001 |
| III | 2.83 | 2.22-3.60 | | <0.001 |
| IV | 3.82 | 3.16-4.27 | | <0.001 |
| Histological grade |  |  | |  |
| Grade I | reference |  | |  |
| Grade II | 1.35 | 1.05-1.73 | | 0.02 |
| Grade III | 2 | 1.56-2.57 | | <0.001 |
| Grade IV | 4.61 | 2.22-9.57 | | <0.001 |
| Surgery |  |  | |  |
| no surgery | reference |  | |  |
| local tumor destruction | 0.47 | 0.30-0.73 | | <0.001 |
| hepatectomy | 0.29 | 0.25-0.34 | | <0.001 |
| transplantation | 0.29 | 0.16-0.49 | | <0.001 |
| Regional lymph surgery |  |  | |  |
| none | reference |  | |  |
| 1 to 3 removed | 0.62 | 0.52-0.74 | | <0.001 |
| 4 or more removed | 0.61 | 0.50-0.74 | | <0.001 |
| Sequence of systemic and surgery |  |  | |  |
| no and/or surgical procedures | reference |  | |  |
| before surgery | 0.64 | 0.46-0.90 | | 0.01 |
| after surgery | 0.61 | 0.52-0.72 | | <0.001 |
| both before and after | 0.92 | 0.49-1.17 | | 0.78 |
| Tumor size |  |  | |  |
| <5cm | reference |  | |  |
| 5~10cm | 1.77 | 1.73-2.60 | | <0.001 |
| >10cm | 2.12 | 1.52-2.07 | | <0.001 |
| Chemotherapy |  |  | |  |
| no/unknown | reference |  | |  |
| yes | 1.67 | 1.44-1.95 | | <0.001 |
| Sequence number |  |  | |  |
| One primary only | reference |  | |  |
| 1st of 2 or more primaries | 0.38 | 0.27-0.54 | | <0.001 |
| not 1st primary | 0.74 | 0.62-0.88 | | <0.001 |
| Total number |  |  | |  |
| one | reference |  | |  |
| two | 0.64 | 0.54-0.77 | | <0.001 |
| three or more | 0.65 | 0.48-0.88 | | <0.01 |
| Months from diagnosis to treatment |  |  | |  |
| >3 | reference |  | |  |
| 0~1 | 1.32 | 1.02-1.70 | | 0.04 |
| 2~3 | 1.10 | 0.84-1.44 | | >0.05 |

**Table S2:**

**Results of multivariate Cox analysis in the training cohort.**

| **Characteristics** | **HR** | **95%CI** | **P Value** | |
| --- | --- | --- | --- | --- |
| Sex |  |  |  | |
| female | reference |  | |  |
| male | 1.16 | 1.01-1.34 | | 0.03 |
| TNM Stage |  |  |  |  |
| I | reference |  | |  |
| II | 1.84 | 1.47-2.30 | | <0.001 |
| III | 3.01 | 2.34-3.89 | | <0.001 |
| IV | 2.74 | 2.20-3.42 | | <0.001 |
| Histological grade |  |  | |  |
| Grade I | reference |  | |  |
| Grade II | 1.22 | 0.95-1.57 | | 0.12 |
| Grade III | 1.37 | 1.06-1.77 | | 0.02 |
| Grade IV | 2.52 | 1.18-5.36 | | 0.02 |
| Surgery |  |  | |  |
| no surgery | reference |  | |  |
| local tumor destruction | 0.86 | 0.50-1.48 | | 0.58 |
| hepatectomy | 0.41 | 0.30-0.57 | | <0.001 |
| transplantation | 0.42 | 0.22-0.79 | | <0.01 |
| Regional lymph surgery |  |  | |  |
| none | reference |  | |  |
| 1 to 3 removed | 1.01 | 0.80-1.26 | | 0.96 |
| 4 or more removed | 0.99 | 0.79-1.26 | | 0.96 |
| Sequence of systemic and surgery |  |  | |  |
| no and/or surgical procedures | reference |  | |  |
| before surgery | 0.86 | 0.54-1.37 | | 0.52 |
| after surgery | 0.78 | 0.55-1.10 | | 0.16 |
| both before and after | 1.20 | 0.59-2.45 | | 0.61 |
| Tumor size |  |  | |  |
| <5cm | reference |  | |  |
| 5~10cm | 1.06 | 0.85-1.33 | | 0.62 |
| >10cm | 1.25 | 1.06-1.48 | | <0.01 |
| Chemotherapy |  |  | |  |
| no/unknown | reference |  | |  |
| yes | 0.72 | 0.60-0.86 | | <0.001 |
| Sequence number |  |  | |  |
| One primary only | reference |  | |  |
| 1st of 2 or more primaries | 0.38 | 0.27-0.54 | | <0.001 |
| not 1st primary | 0.93 | 0.78-1.12 | | 0.45 |
| Months from diagnosis to treatment |  |  | |  |
| >3 | reference |  | |  |
| 0~1 | 1.45 | 1.11-1.90 | | <0.01 |
| 2~3 | 1.14 | 0.86-1.50 | | 0.36 |

**Table S3:**

**Specific parameters of four machine learning models.**

| **Model** | **Parameter** | **Grid Search Space** | **Value** |
| --- | --- | --- | --- |
| CPH | all | NA | default |
| GBM | min_samples_split | (2, 21, dtype=int) ^a^ | 8 |
|  | n_estimators | (50, 351, 10) ^b^ | 290 |
|  | min_weight_fraction_leaf | (0.0, 0.51, 0.1) | 0.1 |
|  | Others ^c^ | NA | default |
| Tree | min_samples_split | (2, 21, dtype=int) | 17 |
|  | min_samples_leaf | (1, 21, dtype=int) | 17 |
|  | Others | NA | default |
| RSF | n_estimators | (80, 151, 10) | 140 |
|  | min_samples_leaf | (1, 21, dtype=int) | 2 |
|  | min_samples_split | (2, 21, dtype=int) | 10 |
|  | others | NA | default |

^a^ “(2, 21, dtype=int)” means that the search interval is [2,21) and only integers are taken

^b^ “(50, 351, 10)” means that the search interval is [50,351], starting at 50, with intervals of 10 values.

^c^ “others” refers to other parameters not mentioned in the table and takes default values.

**Table S4:**

**The performance of internal test cohort and external test cohort in random forest model.**

| **Model** | **Internal test queue** | | **External test queue** | |
| --- | --- | --- | --- | --- |
|  | **C-index** | **Brier Score** | **C-index** | **Brier Score** |
| RSF model | 0.723 | 0.175 | 0.799 | 0.130 |
| Random | 0.500 | 0.265 | 0.500 | 0.196 |
