# Supplementary material for: Development of machine learning models for patients in the high intrahepatic cholangiocarcinoma incidence age group
Source: BMC Geriatr. 2024 Jun 25;24:553. doi: 10.1186/s12877-024-05154-3 (PMC11197277; doi:10.1186/s12877-024-05154-3)
Supplement: Supplementary file 3 — Supplementary Material 3 [file 12877_2024_5154_MOESM3_ESM.docx]

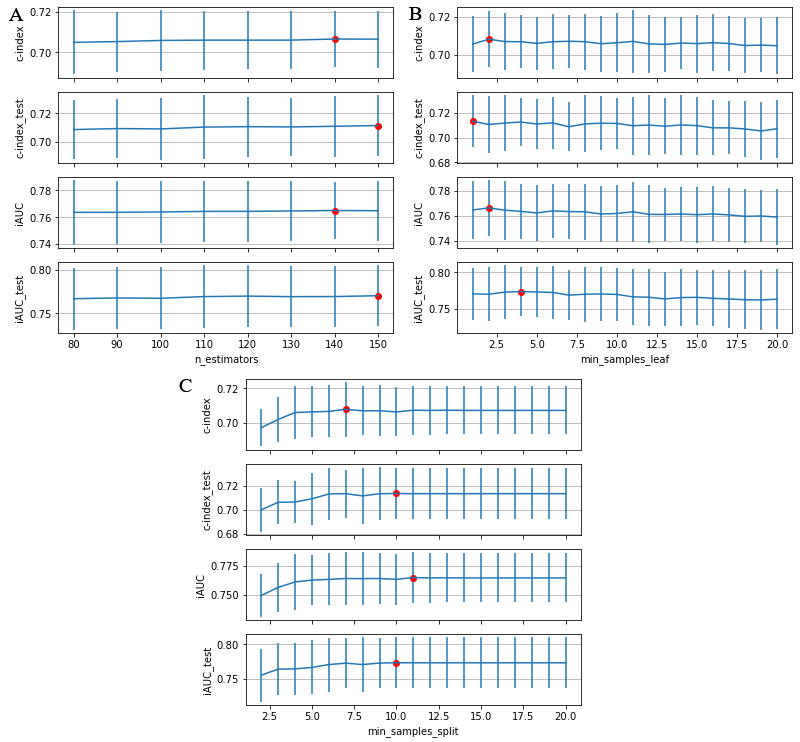


Figure S1. Parameter optimization of RSF model with 5-fold cross-validation. Visualization of grid search for parameters “n_estimators” (A), “min_samples_leaf” (B) and “min_samples_split”.
